# Gender Differences in Depression: Evidence From Genetics

**DOI:** 10.3389/fgene.2020.562316

**Published:** 2020-10-15

**Authors:** Lihong Zhao, Guanghong Han, Yinghao Zhao, Yang Jin, Tongtong Ge, Wei Yang, Ranji Cui, Songbai Xu, Bingjin Li

**Affiliations:** ^1^Jilin Provincial Key Laboratory on Molecular and Chemical Genetics, Second Hospital of Jilin University, Changchun, China; ^2^Department of Oral Geriatrics, Hospital of Stomatology, Jilin University, Changchun, China; ^3^Department of Neurosurgery, First Hospital of Jilin University, Changchun, China

**Keywords:** depression, gender difference, genetics, gene-environment interactions, heritability

## Abstract

Compared with men, female accounts for a larger proportion of patients with depression. Behavioral genetics researches find gender differences in genetic underpinnings of depression. We found that gender differences exist in heritability and the gene associated with depression after reviewing relevant research. Both genes and gene-environment interactions contribute to the risk of depression in a gender-specific manner. We detailed the relationships between serotonin transporter gene-linked promoter region (5-HTTLPR) and depression. However, the results of these studies are very different. We explored the reasons for the contradictory conclusions and provided some suggestions for future research on the gender differences in genetic underpinnings of depression.

## Introduction

Depression is a prevalent mental illness that seriously affects physical and mental health (Krishnan and Nestler, 2008; Ge et al., 2018; Ren et al., 2020). Women are more likely to suffer from depression (Young et al., 1990; Harkness et al., 2010; Wang et al., 2019). The susceptibility to depression is affected by diverse hereditary, epigenetic, environmental, and endocrine risk factors (Duman et al., 2016). With the rise of developmental behavioral genetics (using the research methods and techniques of psychology and behavioral genetics to examine the influence of genetics and environment on the development of human psychology and behavior), more and more researchers began to pay attention to the role of genetic factors in the occurrence of gender differences in depression. Behavioral genetics research methods include quantitative genetics (mainly through twins and adoption research to find evidence that genetics and the environment affect human psychology and behavior) and molecular genetics [identify susceptibility genes associated with specific psychology and behavior, including candidate gene association studies and genome-wide association studies (GWAS)]. Twin studies show differences in the heritability of depression between men and women, and molecular genetics studies show gender differences in depression caused by specific genes and their interaction with the environment. However, these findings are not consistent.

This manuscript reviews relevant studies on the genetic underpinnings of gender differences in depression. Besides, we explored the reasons for the contradictory conclusions and provided some suggestions for future research on the genetic underpinnings of gender differences in the depression. We hope this manuscript will help scientists better understand and study genetic underpinnings of gender differences in the depression.

## Epidemiology

Many national and international studies display that sex ratio (women: men) of depressive disorders over 1.7 for lifetime prevalence and 1.4 for 12-month prevalence after the age of 18 (Kuehner, 2017). The gender difference in depression rates first emerge in adolescence and continues into old age (Angold and Worthman, 1993), although the gender gap of the adult is smaller than it is at younger ages (Patten et al., 2016; Kiely et al., 2019). Similar gender differences exist in different income countries, although significant cross-national variation exists (Van de Velde et al., 2010). But, gender differences do not exist across all race-ethnic groups (Kessler, 2003; Yancu, 2011). The female predominate in the incidence of depressive disorders; instead, there appears to be no gender difference in recurrence, remission, or chronicity of depression (Kessler, 2003; Otte et al., 2016). The symptom profile of men and women with depression is different. Women are more likely to show increased appetite, hypersomnia, somatic symptoms, etc. (Piepenburg et al., 2019). Especially, comorbidity of peripartum depression with anxiety disorders, obsessive-compulsive disorder, and post-traumatic stress disorder worth attention (Kuehner, 2017).

## Gender Differences in Heritability

The family pedigree study finds depression is hereditary. According to reports, children of depressed parents have increased symptoms of depression and internalization (Rice et al., 2002). Later, the twin study divided the sources of phenotypic variation of depression into three aspects: genetic, shared environment, and non-shared environment, which provided the possibility of separating the role of genetic and environmental.

Most Scholars use the twin paradigm in quantitative genetics to investigate gender differences in the genetic basis of depressive symptoms. Research on gender differences in heritability of depressive symptoms mainly focuses on adolescents in European and American countries. Adolescence is a particularly good time when many people will experience the first onset (Eley et al., 2004). During adolescence, the prevalence rate of depression in men and women has begun to rise dramatically, especially in girls. Similarly, the heritability of depression increased from childhood to adolescents (Ksinan and Vazsonyi, 2019). Biological and pubertal changes, cognitive maturity occurs during adolescence, some genetic factors may be “switched on” to promote these changes, which in turn affect depressive symptoms (Lau and Eley, 2006). Jacobson and Rowe (1999) show that the heritability in depressed mood is higher in female adolescents than in male adolescents (self-rated depressive symptoms), however, Rice et al. (2002) shows the opposite result (self-rated depressive symptoms). McCaffery et al. (2008) reported that non-shared environment and the genetic factors contribute to the correlation of depressive symptoms in female adolescents and cigarette smoking; but In male adolescents, only non-shared environment. In an older twin study, the heritability of women was also higher than that of men, although no statistically significant (Jansson et al., 2004). Scourfield et al. (2003) show higher heritability for young girls (children) than young boys only from parent-rated depressive symptoms, not self-rated depressive symptoms. Some methodological differences exist in these surveys, including measurement methods, source of information (informant), the age range of the sample, number of samples, sibling-pairs sample, demographic characteristics (Table 1), which limits comparability between surveys. We are not sure whether the difference in the heritability of depressive symptoms exists between gender.

**TABLE 1:** Gender differences in heritability.

Sibling-Pairs Sample	Age Group	Methodology	Measurement instruments	Demographic Characteristics	Gender composition	Result	Source of information (informant)	References
2,302 pairs Sibling-Pairs	16 years (range = 11–20 years)	Cross-sectional Study	Depressed mood: Center for Epidemiological Studies-Depression (CES-D)	Caucasian, African American, other Ethnicity (A smaller percentage)	Female: 2285, Male: 2319	Heritability in depressed mood is higher in female adolescents than in male adolescents. Genetic factors were higher for female adolescents than male adolescent in correlations between family and school environment and adolescent depressed mood	Self-report	Jacobson and Rowe, 1999
959 twin pairs (123 female MZs, 90 male MZs, 207 same-sex female DZs, 109 same-sex male DZs, and 430 opposite-sex DZs)	50 years or older (mean age 72 years)	Cross-sectional Study	Depressive symptoms: Center for Epidemiological Studies-Depression (CES-D) and self-reported use of antidepressant medication.	Caucasians	Female: 1090, Male: 828	Higher heritability for women than men (no statistically significant).	Self-report	Jansson et al., 2004
287 MZ (143 male-male, 144 female-female pairs) and 441 DZ twin pairs (132 male-male, 113 female-female, and 196 male-female)	Mean age 16.1 years	Cross-sectional Study	Depressive symptoms. Center for Epidemiologic Studies–Depression Scale (CES-D)	Caucasian, African American, Hispanic/Latino other ethnicities (A smaller percentage)	Female: 710, Male: 746	In female, non-shared environment and genetic factors contribute to the correlation of depressive symptoms and cigarette smoking. In male, only non-shared environment.	Self-report	McCaffery et al., 2008
670 twin pairs (MZ and DZ)	5–17 years	Cross-sectional analyses longitudinal study	Depressive symptoms: Parent and self-report questionnaire data Mood and Feelings Questionnaire.	Wales	Female: 636, Male: 612	Only parent-report data show that girls show greater genetic effects than boys.	Self-report	Scourfield et al., 2003
1463 families	8–17 years	Cross-sectional analyses	Depressive symptoms: Mood and Feelings Questionnaire and Hospital Anxiety and Depression Scale	South Wales and Greater Manchester		For self-rated depressive symptoms, adolescents (11 years and over) show greater genetic effects than female	Self-rated parent-rated	Rice et al., 2002
508 MZ, 176 DZ	10–19 years	Longitudinal study	Depressive symptoms: Children’s Depression Inventory, CDI	Chinese		No gender difference in the heritability of adolescent Depressive symptoms	Self-rated parent-rated	Hou et al., 2012

Several reasons can explain the divergence of the above conclusions. First, the genetic factors on depressive symptom vary according to the individual’s developmental stage (such as childhood and adolescence) or age: Both self-reports and parent reports show that individuals with early adolescence have a higher heritability in depressed mood than individuals with mid-adolescence (Hou et al., 2012); genetic factors become more important from childhood to adolescence or less important (Rice et al., 2002; Scourfield et al., 2003). Most studies have analyzed adolescents at different developmental stages of adolescence and may have overlooked the change in genetic interpretation of depressive symptoms during adolescence. Like most complex behaviors, depression does not simply follow Mendel’s single gene inheritance law but is affected by multiple genes, known as quantitative trait locus (QTL). Different genes are turned on in different time, the interaction between genes and the interaction between genes and the environment show different patterns at different stages of development, so the influence of genetics and environment on adolescents’ depression is dynamically changing (Hou et al., 2012). Second, The inheritance rate varies according to the reporter and genetic influences may be less important for child-rated depression symptoms than for parent-rated symptoms (Rice et al., 2002): Proxy ratings can be influenced by the informant’s symptoms of depression and anxiety; Self-reports and parental reports may have evaluated different aspects of depressive symptom or depressive symptom at different moments; in parents-report, parents need to rate two twins. In this process, two twins will be inevitably compared with each other, or the two children will be rated more similarly, or the rating will be less similar. In self-reporting, a child only needs to report themselves’ emotional experience. Third, the small number of subjects may not be sufficient to produce convincing results. Modest heritability (30–40%) (Sullivan et al., 2000), clinical heterogeneity and complicated genetic architecture for major depression requires a larger sample size. In order to generate replicable and statistically significant findings, 75,000–100,000 major depressive disorder cases are needed in GWAS to identify gene loci involved major depressive disorder (Duman et al., 2016). Also, maximizing sample sizes is more informative to understand genetic heterogeneity of depression (Hall et al., 2018).

## Gender Differences in the Gene Associated With Depression

The twin studies found that the genetic factor affect depressive symptom of adolescents but gender difference in heritability of depressive symptom remains to be further studied. Molecular genetics attempts to locate the genes for gender differences in depression. At present, most candidate gene association studies have examined the relationship between serotonin system genes, dopamine system genes and depression (Table 2): loci implicated in the serotonin (5HT) system including serotonin (5-HT) transporter gene-linked promoter region (5-HTTLPR), 5HT receptor 2A (5HT2A), 5HT receptor 2C (5HT2C), monoamine oxidase type A (*MAOA*), tryptophan hydroxylase (*TPH1*). loci implicated in the dopamine system including catechol-*O*-methyltransferase (*COMT*), dopamine receptor genes *DRD1-DRD5*. Related candidate genes can regulate the level of neurotransmitters (serotonin or dopamine) in the synaptic space through degradation (e.g., *MAOA*, *COMT*) and transport (such as 5-HTTLPR), and can also change the number of receptors in the brain (5HT2A, *DRD2* gene) to regulate signal transmission, which in turn affects the level of individual depression.

**TABLE 2:** 5-HTTLPR alone and interaction with the environment contribute to the risk of depression.

Age	Gene	Measurement instruments	Environmental factor	Gender composition	Demographic Characteristics	Source of information (informant)	Study Design	Result	References
337 adolescents aged 10–20 years	5-HTTLPR (L/S), HTR2A, HTR2C, MAOA (monoamine oxidase type A) and tryptophan hydroxylase (TPH)	Short form of Mood and Feelings Questionnaire (SMFQ)	Family environmental risk: family social adversity; parental educational level; adverse life events	Female: 220, Male: 117		Self-report questionnaire	Cross-sectional Study	1. The main effect of 5HTTLPR “short” alleles was significant only in the female. an overall decrease in odds of depression for an increasing number of “short” alleles. 2. 5-HTTPLR “short-short” genotype interacts with high environmental risk increase depression risk only for female	Eley et al., 2004
16–19 years	5-HTTLPR (L/S)	[Depression Self-Rating Scale (DSRS)] of the DSM-IV	1. Type of residence 2. Separated families’ 3. Traumatic conflicts within the family	Female: 11, Male: 81	Swede	Self-reports interview	Cross-sectional Study	1. Boys and girls carrying the short 5-HTTLPR allele react to different kinds of environmental factors. 2. Females rather than male carrying the short 5-HTTLPR allele tended to develop depressive symptoms with the environmental stress factor	Sjöberg et al., 2006
Study 1 288 participants mean age 58.3 Study 2 142 participants Mean age 34.0	5-HTTLPR (L/S)	Study 1 depressive symptomatology: Center for Epidemiologic Studies Depression Scale (CES-D) Study 2 depressive symptoms: The 40-item Obvious Depression scale (OBD)	Study 1 the stressor of caregiving status Study 2 childhood socioeconomic status (SES)	Study 1 215 females Study 2 64 females	Study 1 70.5% Caucasians Study 2 47.2% Caucasians	Study 1 home visit self-reports. Study 2 self-reports.	Cross-sectional Study	1. For females, the s allele, combined with caregiving stress (Study 1) or low childhood SES (Study 2), was associated with higher depression scores as compared to participants in the non-stressor group and those with the long (l) allele. 2. In males, the l allele, combined with a stressor, was associated with higher depression scores as compared to those in the non-stressor group and those with the s allele	Brummett et al., 2008
Between the ages of 22 and 26 (*n* = 4724)	5-HTTLPR (L/S)	Depression: using responses from two questions; depression symptoms (CES-D)	1. Stressful life events 2. Childhood maltreatment.	Male *n* = 2312, Female *n* = 2412	Non-Hispanic white	self-reported	Cross-sectional Study	1. 5-HTTLPR plays a role in moderating the impact of SLEs on depression status, a statistically significant only in males (for SS genotype). 2. For females carrying one or more of the S-alleles, the prevalence of suicide ideation increased with an increasing number of stressful life events. whereas, for males, the prevalence rates increased for carrying one or more L-alleles	Haberstick et al., 2016
Students 17–18 years	5HTTLPR (L/S)	Self-rating scale (DSRS) of the DSM-IV	Maltreatment	Male *n* = 765, Female *n* = 717	Scandinavian 1245 Non-Scandinavian 217	self-reported	Cross-sectional Study	A significant main effect and a G × E interaction effect of the SS allele was found only among girls.	Aslund et al., 2009
346 youth mean age 23.7 years	5HTTLPR (L/S)	Depressive symptoms: Beck Depression Inventory−II	Negative acute life events Chronic family stress	132 males, 214 females	93% Caucasian	Interview measures	Longitudinal Study	A significant interaction between family discord and genotype only among females. The effect of family discord on BDI was stronger in SL and SS females compared to LL females	Hammen et al., 2010
In males: 12–19 years; In females: 12–20 years	5−HTTLPR (L/S)	Depressive symptom: Epidemiological Studies Depression Scale (CES−D)	Family structure Family−level socioeconomic status (SES) Social support County−level environment	Females (*n* = 560), Males (*n* = 524)	White (reference), African−American, Hispanic, Asian, and other race	In-home interview self-report	Longitudinal Study	1. Among females, the main effects models showed an association between the SL genotype and lowered risk of depressive symptoms. 2. Among males, interaction models showed an association between SL genotype and lowered risk of depressive symptoms in deprived counties only	Uddin et al., 2010
12–19 years, males; 12–20 years, females	5-HTTLPR (L/S)	Depressive Symptom: 17-item version of the Center for Epidemiological Studies Depression (CESD)	1. Respondent-level building conditions 2. Neighborhood-level building conditions	1. Male (*n* = 510) Female (*n* = 574) 2. Male (*n* = 377) and Female (*n* = 418)	White (reference), African American, Hispanic, Asian, and other races.	Self-reported	Cross-sectional Study	1. No gene-social environment interaction effects 2. Respondent-level building analyses provided some evidence for genetic influences on depressive symptom score in adolescent females 3. Neighborhood-level building analyses provided evidence for increased depressive symptom score among adolescent males only residing in neighborhoods with poorer building conditions, in both unadjusted and adjusted results.	Uddin et al., 2011
	5-HTTLPR (L/S) monoamine oxidase A-upstream variable number tandem repeat (MAOA-uVNTR)	Depressive Symptoms: Children’s Depression Inventory (CDI)	Negative life events (NLE)	129 female, 180 male	89.3 % were White, 1.7 % African American, 1.7 % Hispanic, 1.2 % American Indian/Alaskan, and 6.15 % biracial or multiracial.	Self-reported	Longitudinal Study	1. Girls were most likely to exhibit elevated depressive symptoms when experiencing NLE if they possessed low-expression MAOA-uVNTR alleles and short 5-HTTLPR alleles 2. Low-expression MAOA-uVNTR alleles but long 5-HTTLPR alleles were implicated in boys at the age of 13	Priess-Groben and Hyde, 2013
	Brain-derived neurotrophic factor (BDNF) val66met and the serotonin transporter region 5-HTTLPR (L/S),	depressive symptoms: Beck Depression Inventory-II (BDI-II)	Family environment quality	140 males, 223 females	92% White, 1.5% Asian, 6% biracial, and 0.5% other/not reported	Interview and self-report	Longitudinal Study	After age 15, the interaction of cumulative plasticity genotype (defined as presence of neither, either, or both 5-HTTLPR S and val66met Met alleles) and early family environment quality was only predictive of depression among females	Dalton et al., 2014
The average age 15.5 years	5-HTTLPR (L/S)	Depression symptoms: 17-item version of the Center for Epidemiologic Studies Depression Scale (CES-D;	Family support	56% of boys	Caucasian	Self-report in-home interview	Cross-sectional Study	In the presence of poor family support, boys with at least one short allele had more symptoms of depression. in the presence of high family support, boys with the short allele had the fewest depression symptoms	Li et al., 2013
Mean age 38.3 ± 10.3 years	A tri-allelic serotonin transporter promoter polymorphism (5-HTTLPR/rs25531) low-expressing tri-allelic analyses, S’(S, L_*G*_) and high-expressing L’(L_*A*_, XL_*A*_) bi-allelic analyses (L/S)	Beck Depression Inventory (BDI) Maudsley Personality Inventory (MPI)		550 men, 589 women	Healthy Han Chinese	Self-report	Cross-sectional Study	1. Tri-allelic genotype-by-gender interaction: S′S′ homozygotic were associated with higher neuroticism and BDI scores in men. 2. Women showed a non-significant pattern across both the 5-HTTLPR classifications 3. In the bi-allelic analyses, there was only an association between SS genotype and MPI-neuroticism in men	Chang et al., 2017
Aged from 14 to 18	5-HTTLPR (L/S)	Depressive symptoms: Center for Epidemiological Studies Depression Scale (CES-D)	Negative life events	131 females and 121 males	Chinese healthy Han population	Self-report interview	Longitudinal Study	No main effect of 5-HTTLPR A significant 5-HTTLPR × stress interaction in females only. Females with at least one 5-HTTLPR S allele exhibited more depressive symptoms under stressful situations. No significant 5-HTTLPR × stress interaction was found in males	Ming et al., 2013

**FIGURE 1:**
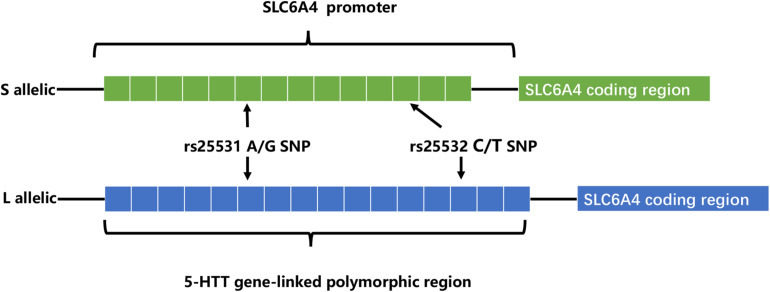
SLC6A4 L and S allelic.

Recently, extensive works of literature have investigated the relationships between 5-HTTLPR and depression, the serotonin transporter gene-linked promoter region (5-HTTLPR) is a variable number tandem repeats (VNTR) located in the promoter region of *SLC6A4* (the human 5-HTT-encoding gene) (Iurescia et al., 2016). In addition to most common alleles: the short (S, 14 repeats) and the long (L, 16 repeats), there are less common alleles: extra-long (XL, 17–24 repeats) and extra-short (XS, 11–13 repeats). The L allele possesses higher transcriptional activity and serotonin uptake rate than S allele positively affects serotonin reuptake rate. Also, two nearby single nucleotide polymorphisms (SNPs) rs25531 and 25532 (located in the 5-HTTLPR) contribute to the functional variations of *SLC6A4* expression (Iurescia et al., 2016; Figure 1). The 5-HT transporter (5-HTT), an integral membrane protein, moves 5-HT from synaptic space into presynaptic neurons (Damsbo et al., 2019; Möller et al., 2019). And then 5-HT was degraded by MAOA or recycled into synaptic vesicles. Duration and magnitude of 5-HT biological actions are closely related to 5HTT (Coleman et al., 2019). Also, effective drugs selective serotonin reuptake inhibitors (SSRIs), act on 5-HT transporter (Ananth et al., 2018; Kulikov et al., 2018). So, dysfunction in 5HTT leads to psychiatric disorders including depression.

### Genes Directly Affect Depression in a Gender-Specific Manner

Different genes may directly affect depression in a gender-specific manner. 5HT2A, TPH may be a risk gene for depression in women, and COMT may have a greater impact on men. The relationship between 5-HTTLPR genotype and depression is highly controversial: although females carrying short alleles had a lower risk of depression than other genotypes (Table 2), these research results show inconsistent conclusions on specific genotypes (Eley et al., 2004; Aslund et al., 2009; Uddin et al., 2010). Animal studies have shown that individuals carrying short alleles, especially female animals, are more vulnerable to chronic stressors (Spinelli et al., 2012). But, Others showed no main effect of 5-HTTLPR on depression, which means 5-HTTLPR genotype cannot predict depression risk (Aslund et al., 2009; Risch et al., 2009; Ming et al., 2013). The study also indicated the main effects of 5HT2A, TPH on depression group exist in female subjects only (Eley et al., 2004). However, another study found direct effects of certain depression-related genes only exist in the male population. Individuals carrying the Met/Met of COMT genotype are less likely to suffer depression than those carrying the Val/Val genotype (Baekken et al., 2008).

In addition to candidate gene association studies, GWAS is another research strategy in the field of molecular genetics to find genes associated with individual psychological or behavioral phenotypes. Recent GWAS has identified 14 independent and replicated loci that were associated with MDD at the genome-wide level (Maul et al., 2020). Only a few scientists have reported gene loci related to gender differences in depression: SNP rs6602398, presented in interleukin receptor 2A gene (IL2RA), was significantly associated with males MDD (Powers et al., 2016); 2 SNPs rs619002 and rs644926, presented in the EH-domain containing 3 (EHD3) gene, were associated with female MDD (Wang et al., 2014). However, some scientists showed no evidence for genetic heterogeneity between the gender using GWAS summary statistics (Trzaskowski et al., 2019).

Candidate gene association studies and Genome-wide association studies (GWAS) are research methods in developmental-behavioral genetic, aiming to find out whether genetics and environment affect human psychology and behavior development. Candidate gene association research is to directly select genes that may be related to individual psychological or behavioral phenotype variation based on existing genetic related information, biological related information, or empirical research results and then to determine whether a candidate gene is associated with this phenotype by case-control study or population-based association analysis. GWAS selects SNPs associated with individual psychological or behavioral phenotypes from sequence variations (single nucleotide polymorphism, SNP) throughout the human genome. The difference from candidate gene research strategies is that you do not need to know the function and characteristics of genes in advance. Also, there are no preset research assumptions. It offers opportunities for finding unknown susceptibility genes. Though more and more depression loci are identified, most GWAS has not yet made a replicable discovery of MDD (Hyde and Mezulis, 2020). Also, the GWAS study of depression has not achieved the same success as other mental illnesses; the complexity of the genetics and phenotype of depression may mean that a GWAS study will require a sample of thousands of participants (Howard et al., 2019). Compared with the huge cost of GWAS, candidate gene association studies are more economica and faster.

### Gene-Environment Interaction Contribute to the Risk of Depression

Many studies suggest 5HTTLPR-negative environment interaction contributes to the risk of depression in the child, adolescent, and adult populations in a gender-specific manner (Table 2). Also, sex modulates 5-HTTLPR genotype-childhood adversity interaction on hippocampal volume [reducing hippocampal volume in depressed patients (Maller et al., 2018)] (Everaerd et al., 2012). But, results remain inconclusive. Some studies have shown females rather than males carrying the SS genotype of 5-HTTLPR tended to develop depressive symptoms under negative environment (Eley et al., 2004; Aslund et al., 2009; Hammen et al., 2010; Ming et al., 2013) or females carrying S allele are easier to develop depressive symptoms under negative environment (Sjöberg et al., 2006; Brummett et al., 2008; Hammen et al., 2010; Ming et al., 2013). However, many contradictions about 5HTTLPR-negative environment exist in males: the l allele-stressor interaction contributes to higher depression scores as compared to those control group and s allele (Brummett et al., 2008); Uddin et al. (2010) showed an interaction between SL genotype and deprived counties predicted lowered risk of depressive symptoms in males; Li et al. (2013) showed the interaction between poor family support and SL genotype predicted more symptoms of depression in males; Other studies showed SS genotype-negative environment interaction predicted higher risks of depression, a statistically significant only in males (Li et al., 2013; Haberstick et al., 2016; Chang et al., 2017). Basically consistent conclusion exist in the females but not males. Under negative environment, females carrying S alleles have higher depression levels. But, A Meta-Analysis of Interaction between 5-HTTLPR, stressful life events, and risk of depression, published in 2009, neither 5-HTTLPR genotypes alone or interaction with stressful life events predicted an increased risk of depression in females alone, males alone, or in both genders combined. The Meta-Analysis across 14 studies, subjects of most studies are adults (Risch et al., 2009).

**FIGURE 2:**
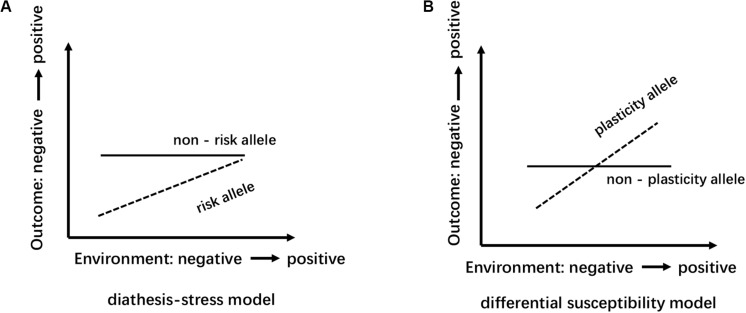
Schematic diagram of the model of differential susceptibility and diathesis-stress: the abscissa represents the transition of environment factors from negative to positive; the ordinate indicates that the outcome variable range from negative to positive. **(A)** Diathesis-stress model points out that individuals with “risk” allele are only more likely to be negatively affected and to develop poorly than those with the “non-risk” allele. **(B)** Differential susceptibility model view, compared with the “non-plastic” allele carriers, “plastic” alleles individual has better sensitivity, more sensitive to both positive and negative environment accordingly develop better or worse.

Several reasons can explain the divergence of the above conclusions. First, Different countries and races have different distributions of alleles and genotypes of the 5-HTTLPR: e.g., different frequency of S/S and L/L genotype between older Taiwanese adults and western groups (Goldman et al., 2010); the higher frequency of S alleles in Asians than in Caucasians (Iurescia et al., 2016). Second, the dichotomous classification (S/L) of 5-HTTLPR genotypes may lead to influenced research results. Increased length of the 5HTTLPR may be associated with increased gene expression (S < L < XL) (Vijayendran et al., 2012). But, dichotomous classification of 5-HTTLPR genotypes exists in most studies (Table 2). Third, neglecting the two nearby SNPs rs25531 and 25532 may lead to a contradictory conclusion. SNP rs25531 contributes to different allelic subtypes S_A_, S_*G*_, L_A_, and L_*G*_. The different expression abilities exist in L_A_/L_A_ genotype AND S/S, S/L_*G*_, L_*G*_/L_*G*_, L_A_/L_*G*_. Fourth, gene-environment interaction may be more successful for studies that study a single gene with big environmental impact. For example, uninfected control group subjects, carrying 32 mutation in the ΔCCR5 chemokine receptor, were less infected with human immunodeficiency virus when they were highly exposed to the virus (Risch et al., 2009). However, The inheritance of depression does not follow a single-gene inheritance pattern like Huntington’s disease but has a non-Mendelian, polygenic underpinning. As a complex psychological problem, depression is most likely the result of the synergistic effects of multiple genetic and environmental factors (Cao et al., 2018). In recent years, the studies of polygenic risk scores and gene-gene interaction studies have proved additive and interactive genetic effects of depression. Also, multi-genes affect the development of depression through interaction with environmental factors and gender differences exist in this complex interaction (Cao et al., 2016). e.g., Girls rather than boys possessed low-expression MAOA-uVNTR alleles and S 5-HTTLPR alleles, more likely to show increased depressive symptoms under stressful life events (Priess-Groben and Hyde, 2013). The interaction of both plasticity genotype (5-HTTLPR S and val66met Met allele)- early family environment quality predicted more depressive symptoms than either or neither plasticity genotype only in females (Dalton et al., 2014). Fifth, different study design, longitudinal study, and cross-sectional study have their advantages and disadvantages (Table 3). The cross-sectional study is a comparative study of people of different age groups at the same time (intergroup comparison), and the longitudinal study is a continuous study of the same population in various years (self-comparison). Sixth, different gene-environment results between objective measures (i.e., independent of the participants’ report) and subjective measures (i.e., self-report) (Uddin et al., 2011), results of self-reported are more subjective (Sjöberg et al., 2006). Moreover, gene-environment interaction has a dynamic effect on depression. In a study of the influence of BDNF Val66met and 5-HTTLPR on depressive symptoms, Scientists report that the gene-environment interaction conforms to differential susceptibility model when women are 15 years and that gene-environment interaction conforms to the diathesis-stress model after 15 years (Figure 2). Finally, measurement instruments, environmental factors, and source of information (informant) highly divergent across studies, so limiting the comparability and replication of the studies.

**TABLE 3:** Comparison of advantages and disadvantages between longitudinal research and cross-sectional research.

	Longitudinal Study	Cross-sectional Study
Advantages	1. No intergenerational effect 2. Systematically evaluate the behavioral changes of the subjects	1. Higher efficiency of data collection 2. Lower Research and control cost 3. Less affected by the loss of subjects
Disadvantages	1. Time-consuming 2. Higher Research and control cost 3. Loss of subjects brings confounding variables 4. Only observing the changes of one group, whether it has universal significance is still in doubt	1. More confusion variables 2. Can’t systematically evaluate the behavioral changes of the subjects

Besides, a SNP of the *HTR2C* gene, rs6318 (Ser23Cys), is Related to women’s depressive symptoms with high stress levels and different cortisol release (Brummett et al., 2014). related genes of dopamine system (*DRD2*, *COMT*) (Vaske et al., 2009; Nyman et al., 2011), HPA axis system (*CRHR1*) (Roy et al., 2018), and immune system (IL-1β SNP) (McQuaid et al., 2019), can also interact with the environment to affect the occurrence of depression in a gender-specific manner (Table 4). Chinese scientists longitudinally studied the relationship of BDNF Val66Met (Fan et al., 2017), Preproghrelin Leu72Met (Su et al., 2017), oestrogen receptor alpha gene (*ESR1*) rs9340799 (Feng et al., 2017), adiponectin rs1501299 (Wang et al., 2015), tumor necrosis factor receptor-II (TNF-RII) rs1061622 (Memon et al., 2018) and insertion/deletion polymorphism at angiotensin-converting enzyme gene (ACE I/D) (Fan et al., 2018)with depression in adolescents after the 2008 Wenchuan earthquake. Results showed gene-environment interaction contributes to the risk of depression after the earthquake in a gender- and time-dependent manner. Dynamic genetic effects on depression across development were proved once again. However, the scientists also explained that their research is either different from previous research results or rarely reported. Therefore, we cannot yet draw a definitive conclusion on gender differences.

**TABLE 4:** Gene-environment interaction contribute to the risk of depression.

Age	Gene	Measurement instruments	Environmental stress factor	Gender composition	Demographic Characteristics	Source of information (informant)	Methodology	Result	References
Waves II and III (18–27) of the National Longitudinal Study of Adolescent Health (Add Health)	DRD2	Depressive symptoms: Center for Epidemiologic Studies-Depression Scale	Violent Victimization		African American, Caucasian	self-reported in-home interviews	Cross-sectional Study	1. Violent victimization has a strong independent effect on depressive symptoms for Caucasian females. violent victimization is associated with higher levels of depressive symptoms among African American females when they carry at least one A1 allele of DRD2. 2. DRD2 has a significant independent effect on depressive symptoms for males and African American females	Vaske et al., 2009
	Corticotrophin-releasing hormone receptor-1 gene (CRHR1) variant (rs17689918)	Depressive symptoms: Montgomery-Åsberg Depression Rating Scale; mood disorders: Mini-International Neuropsychiatric Interview	Stressful life events (SLE)	52.5% females	European origin	Self- and parent-reports	Longitudinal Study	1. A-allele males and GG females with higher SLEs reported greater depressiveness at age 18 2. Low SLE was associated with a lower risk for depression in males with the GG genotype at age 15	Roy et al., 2018
	SLC6A4, TPH2, COMT, MAOA, and the dopamine receptor genes DRD1–DRD5.	depressive symptoms: HSCL questionnaire	1. Early developmental risk 2. Social environment risk	2509 males, 2716 females	genetically isolated population-based Northern Finland Birth Cohort	Self-report	Longitudinal Study	1. No major genetic effects of the analyzed variants on depressiveness. Rs4274224 from DRD2 shows a significant association with depressiveness in males 2. Allelic variants of COMT interacted with high early developmental risk associated with depression in males	Nyman et al., 2011
Carleton University first-year students	IL-1β rs16944, IL-6 rs1800795 SNP, TNF-α rs1800629	Depressive Symptoms: 21-item Beck Depression Inventory (BDI)	Childhood Maltreatment	343 females and 132 males	various ethnic backgrounds	Self-report	Cross-sectional Study	Among females, higher childhood maltreatment was accompanied by elevated depressive symptoms irrespective of the IL-1β SNP, but among males, this relationship was particularly pronounced for those carrying the GG genotype of the IL-1β SNP.	McQuaid et al., 2019
	HTR2C gene, rs6318 (Ser23Cys)	Depressive symptoms: brief CES-D	Stressful life events	Men 2,366, Women 2,712	White	Self-report	Cross-sectional	Homozygous Ser23 C women who reported high levels of life stress had depressive symptom scores that were about 0.3 standard deviations higher than female Cys23 G carriers with similarly high stress levels.	Brummett et al., 2014
High school (grades 11–12)	BDNF Val66Met	Depression severity: Beck Depression Inventory (BDI)	Wenchuan Earthquake	Males 306, Females 399	Chinese Han	Self-report	Longitudinal Study	1. Females constantly had higher depression prevalence than the males during the follow-up in the Met allele carriers 2. Compared to that at 6 months, the prevalence was lowered at 12 months in the male Met allele carriers, and at 18 months in all the females and the male Met allele carriers.	Fan et al., 2017
High school (grades 11–12)	Preproghrelin Leu72Met	Beck Depression Inventory (BDI)	Wenchuan Earthquake		Chinese Han	Self-report	Longitudinal Study	1. Females had a higher prevalence of depression than males at 6 months after the earthquake in 72Leu/Leu homozygotes 2. The prevalence was consecutively decreased in male 72Met allele carriers, but not in male 72Leu/Leu homozygotes, female 72Met allele carriers, or female 72Leu/Leu homozygotes during follow-up	Su et al., 2017
439 Chinese Han adolescents	Oestrogen receptor alpha gene (ESR1) rs9340799	Beck Depression Inventory (BDI)	Wenchuan Earthquake	Males 197, Females 242	Chinese Han	Self-report	Longitudinal Study	ESR1 rs9340799 maybe not associated with neither the prevalence nor the severity of depression in male individuals, but in female	Feng et al., 2017
Grade 11–12	Adiponectin rs1501299	Beck Depression Inventory (BDI)	Wenchuan earthquake.	Males 233, Females 304	Chinese Han	Self-report	Longitudinal Study	1. The decreases of the scores were found in the male subjects regardless of the genotypes in the time course of 6, 12, and 18 months after the earthquake. 2. The scores were decreased in the female T carriers, but not in the female GG homozygotes at 18 months when compared with those at 12 months after the earthquake.	Wang et al., 2015
High school students	Tumor necrosis factor receptor-II (TNF-RII) rs1061622	Beck Depression Inventory (BDI)	Wenchuan earthquake	Males 197, Females 242	Chinese Han	Self-report	Longitudinal Study	1. Female TT homozygotes had a higher depression prevalence than the male TT homozygotes at 6, 12, and 18 months. 2. The female G allele carriers had a higher depression prevalence than the male G allele carriers only at 6 and 12 months after the earthquake. 3. BDI scores declined in the male subjects with both genotypes and only in the female G allele carriers at 12 months when compared with those at 6 months	Memon et al., 2018
	Insertion/deletion (I/D) polymorphism at angiotensin-converting enzyme gene (ACE)	Depressive symptoms: Beck Depression Inventory (BDI)	Wenchuan earthquake	Males 187, Females 244	Chinese Han	Self-report	Longitudinal Study	1. The D-allele carriers had lower depression prevalence than II homozygotes at 6, 12, and 18 months after the earthquake only in females, but not in males. 2. BDI scores were reduced in the female D-allele carriers when compared with those in the female II homozygotes at 6 and 12 months after the earthquake	Fan et al., 2018

## Future Directions

**FIGURE 3:**
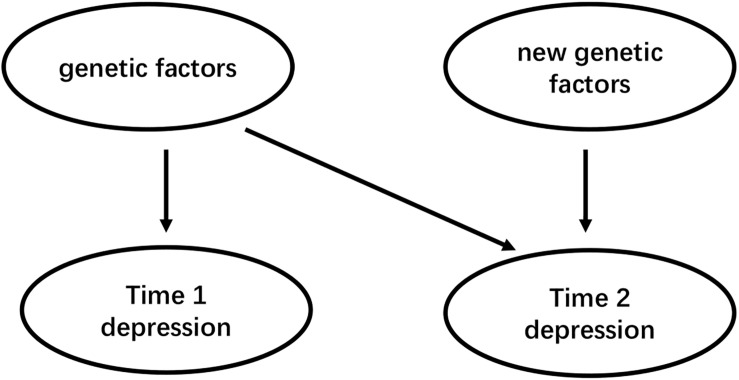
The continuity and variability of genetic factors for depression.

To date, few pieces of research have investigated gender differences in the polygenetic mechanisms of depression, and ignoring gender specificity may lead to inconsistent results. As a complex psychological problem, depression is most likely the result of the synergistic effects of multiple genetic and environmental factors (Cao et al., 2018). Therefore, future studies should further investigate the role of gender in the regulation of polygene genetic mechanisms (Cao et al., 2016). Second, gender differences in the genetic basis of depression may be caused by differences in the sensitivity of individuals to different types of environments (Cao et al., 2013). Future studies should examine the interaction between different types of the environment and genetic genes that affect gender differences in depression. The theoretical basis of the existing molecular genetics research on depression is mostly the “diathesis-stress model” because the many scientists believe that when individuals are under stress or high pressure, psychological and behavioral problems are prone to occur in individuals with a certain type of poor genetic quality, so studies based on this model mostly use the negative environment such as stressful life events as indicators to investigate the G × E effect of depression. However, the newly emerging theoretical model, the “differential susceptibility model,” clearly puts forward and proves that individuals of certain genotypes are also more susceptible to the effects of positive growth environments and perform well or the opposite (Figure 2). Also, the existing research based on the “diathesis-stress model” fails to reveal multiple possible ways of G × E interaction. Whether there is a gender difference in the sensitivity of individuals with different genotypes to the positive environment is also needed for future research. Third, developmental behavioral genetics can investigate in depth whether genetics and the environment have an impact on human psychological and behavioral development and whether the effects were moderated by age. Compared to younger aged youth, older aged adolescents carrying SS/SL genotype has a higher risk of depressive episodes with greater chronic peer stress over the 3 years (Hankin et al., 2015). Besides, Depression is developmentally dynamic and may be affected by some new genetic factors across development (Figure 3). some new genetic factors emerge in depressive symptoms (Lau and Eley, 2006) or symptoms of anxiety and depression (Nivard et al., 2015) in adolescence. Compared with the 5-month-old baby, the negative emotionality of an 18-month-old baby was affected by persistent and new genetic factors (Schumann et al., 2017). So, it is necessary to use a longitudinal cohort design to investigate the gender differences in the genetic basis of depression at different ages and their developmental changes. Forth, Subjects suffering from mental disorders or various physical diseases may rate disease inaccurately (Chang et al., 2017). So, Selecting physically and mentally healthy, drug-free subjects to minimize these confounding factors and reveal the effect of the gene on depression more accurately. Finally, It is worth mentioning that to reduce the interference of confounding factors (e.g., ethnicity, gender, age, socioeconomic status), researchers should add the covariate × environment and covariate × gene interaction terms to the same model that tests the G × E interaction (Keller, 2014).

## Conclusion

There is not enough evidence for genetic heterogeneity in men and women with major depression (Piccinelli and Wilkinson, 2000; Maciej et al., 2019). Genetic markers of major depression have not been successfully identified. Similarly, specific susceptibility genes on the X chromosome have not been successfully identified (Hyde and Mezulis, 2020). As a heterogeneous and multifactorial disease, the gender gap in depression may be caused by many biological, psychological, micro and macro environmental factors with varying interactions (Piccinelli and Wilkinson, 2000; Kuehner, 2017). Heredity may play a role in explaining gender differences. But, no sufficient evidence can explain the gender difference in depression from genetic underpinnings. In future research, scientists should pay attention to the influence of confounding factors on the results. such as different types of environments (positive or negative), demographic characteristics, measurement instruments, study design and so on.

## Author Contributions

LZ wrote the first draft and participated in the discussion of the manuscript. LZ, GH, YZ, YJ, TG, WY, RC, SX, and BL made major revisions to the logic of this manuscript and provided the critical revisions. All authors approved the final version of the manuscript for submission.

## Conflict of Interest

The authors declare that the research was conducted in the absence of any commercial or financial relationships that could be construed as a potential conflict of interest.
